# Exploring the psychometric properties of a tripartite model of risk perception (TRIRISK) in a general U.S. population sample

**DOI:** 10.1080/21642850.2022.2143363

**Published:** 2022-11-10

**Authors:** Destiny Diaz, Liane M. Schneller, Brian V. Fix, Maansi Bansal-Travers, Craig R. Colder, Richard J. O’Connor

**Affiliations:** a Department of Health Behavior, Roswell Park Comprehensive Cancer Center, Buffalo, NY, USA; b Department of Psychology, University at Buffalo, State University of New York, Buffalo, NY, USA

**Keywords:** Tobacco, risk perception, TRIRISK model

## Abstract

**Objective:**

Risk perceptions are key constructs in some theories of health behavior. A tripartite model of risk perception, the TRIRISK model, was developed to assess deliberative, affective, and experiential components of risk perception. The current paper attempts to replicate the factor structure of the TRIRISK measure for cancer and extend the structure to respiratory illness.

**Methods and Measures:**

Participants 18 or older were recruited using an address-based sample in New York State to participate in a Web-based survey. We employed the TRIRISK questionnaire with respect to cancer and respiratory illness. Confirmatory Factor Analyses were conducted in Mplus to validate the TRIRISK model in our sample. TRIRISK model fit across demographic and behavioral groups was tested using multiple-group models.

**Results:**

Of the 704 people included in the analysis, the mean age of participants was 46.9, the majority reported being female (58.5%), and most were White (81.7%). For cancer and respiratory illness, items loaded on the respective constructs as expected. Overall, the TRIRISK model framework fits well across differing subgroups, suggesting that this is a valid model of risk perception to use in a general population sample.

**Conclusion:**

These results provide further evidence that the TRIRISK model is a good model to use for risk perceptions in tobacco control research. The TRIRISK model can be used to communicate risk to encourage positive health behaviors among most sociodemographic groups.

## Introduction

Risk perceptions are key constructs in theories of health behavior wherein individuals make choices by weighing out the risks of consequences with benefits of the action (Ferrer & Klein, 2015). Theory-guided health behavior change interventions and health communications often target risk perceptions with the ultimate goal of changing health behaviors (Ferrer & Klein, 2015). Risk perception has been found to be a multidimensional construct, and while this dimensional structure may be similar across hazards, dimensions of risk could be held at different levels of importance (Wilson et al., 2019). Thus, a multidimensional measure of risk perception may be able to better predict self-protective behavior and decision-making (Wilson et al., 2019). Studying risk perception models can help to advance our understanding of risk perception and how it can be used to influence and target individuals.

Traditionally, risk perception measures focused primarily on cognitive evaluation of risk, though more recent models of risk perception and decision-making have focused on 1) deliberative and 2) affective or experiential components (Ferrer & Klein, 2015). These evolve from dual-systems theories of decision making, which distinguish deliberative from heuristic processes (Chaiken & Trope, 1999; Gerrard et al., 2008; Portnoy et al., 2014; Slovic et al., 2004). *Deliberative* risk perceptions are systematic, logical, and rule-based (Ferrer & Klein, 2015), whereas *affective* risk perceptions refer to the emotional response associated with risk. Worry or anxiety about a threat is considered to be an affective response (Ferrer & Klein, 2015). *Experiential* risk perceptions refer to heuristics (rapid judgments) made by integrating deliberative and affective information (Ferrer & Klein, 2015). Ferrer et al. found that the correlation between the deliberative and experiential factors was high, but discriminant validity was not necessarily in question, based on these correlations and the worse model fit when the latent factors were collapsed into one factor. The TRIRISK model (Ferrer et al., 2016) was developed to assess all three components simultaneously.

Two studies by Ferrer et al (Ferrer et al., 2016) assessed the TRIRISK model across perceptions of risk for cancer, heart disease, and diabetes. Factor analytic tests of the tripartite risk perception structure were done using confirmatory factor analyses. Deliberative, affective, and experiential risk perceptions were assessed by 18 questions (Table 3) derived largely from previously published and validated scales (Ferrer et al., 2016). The tripartite factor structure had a significantly better fit than either dual-construct or single-construct models, and correlations among deliberative, affective, and experiential risk perceptions supported the discriminant validity of these components. The TRIRISK model also showed acceptable concurrent (participants with a diagnosis of cancer showed higher deliberative, affective, and experiential risk perceptions and exhibited more differentiated risk perceptions compared to participants with no such diagnosis), predictive (deliberative, affective, and experiential risk perceptions each significantly and independently predicted intention to protect oneself against cancer), and discriminant (differential interactions observed between the need for affect and the deliberative, affective, and experiential components of risk) validity (Ferrer et al., 2016). Finally, with respect to predicting health behaviors, affective perceptions were the strongest overall predictor, though there were complex interactions among components (Ferrer et al., 2018).

Despite these advances in measurement, the TRIRISK model has not seen widespread adoption, at least as measured by citations in Scopus (75 total since 2016). The measure had also only been examined for three chronic conditions. There are inconsistencies in the literature regarding how smokers perceive their risk, with research concluding over and underestimations. These inconsistencies may stem from the variations in how risk perceptions are measured. Limitations of current measures include that there has been a failure of studies to re-administer previously published multi-item measures and that the measures that are published are not uniform, are not consistent with recommendations, and have not been studied on susceptible populations (Kaufman et al., 2020a; Kaufman et al., 2020b; O'Brien et al., 2019). In this paper, we examine the psychometric properties of this model to provide further evidence for researchers to use, particularly in the area of tobacco control.

To our knowledge, this is one the first studies (besides the original Ferrer et al. paper) that examined the psychometric properties of this measure and extend it to a smoking-related disease. A recent UK study assessed the replicability of the TRIRISK model by CFA, explored the factor structure of risk perception in the UK sample by exploratory factor analysis (EFA), and assessed the associations of EFA-based factors with intentions to change behavior and subsequent behavior change (Riedinger et al., 2022). They confirmed that risk perception is a multidimensional construct and identified self-reflective risk perception as a new distinct component with predictive validity for intention (Riedinger et al., 2022). As part of a statewide survey conducted in New York to examine the impact of flavored tobacco regulations, the TRIRISK measure was included to characterize perceptions of risk around cancer and respiratory illness. The goals of the current paper were to 1) replicate the factor structure of the TRIRISK measure for cancer; 2) extend and verify the structure to respiratory illness; and 3) test whether the overall model is robust to tobacco use status, a key and widely known risk factor for both cancer and respiratory illness.

## Materials and methods

### Participants

Participants could be included in the study if they were residents of New York, 18 or older, who agreed to participate in the study. The survey was developed and conducted between July and October 2020. Address-based sampling was used to recruit survey participants. An invitation letter was sent by mail to these addresses asking the adult in the household with the next birthday to participate via a Web-based survey. Participants were sent a $20 Amazon gift code via email upon completion as compensation. The survey response rate was 3.2%. A total of 946 participants completed the survey; 704 participants are included in these analyses (based on complete responses to the measures of interest). The study protocol was approved by the Roswell Park Institutional Review Board (I-567719).

### Measures

#### TRIRISK questionnaire

We administered the TRIRISK questionnaire with respect to cancer as outlined by Ferrer et al. (2016). This questionnaire also addressed the perceived risk of ‘respiratory illness,’ similar to prior extensions to heart disease and diabetes (Ferrer et al., 2016). We did not define ‘respiratory illness’ *a priori* as a specific condition, such as chronic obstructive pulmonary disease or asthma. Therefore, results should be interpreted relative to this general condition. Response options and scoring were performed as described in prior work (Ferrer et al., 2016).

#### Smoking status

Participants provided information on cigarette smoking status. Smoking status groups were derived and included current cigarette users (participants who reported ‘everyday’ or ‘someday’ use of cigarettes), former cigarette users (participants who reported ‘ever use’, but not current use of cigarettes), and never cigarette users (participants who reported never using cigarettes).

#### Demographic characteristics

Participants also provided information on demographic characteristics. Demographic variables used in this analysis were dichotomized so that we could compare distributions with the Ferrer et al. paper (Ferrer et al., 2016) and so that they could be used in multiple group model analyses. They were dichotomized as follows: sex (male/female), race/ethnicity (White/Other), education (less than college/more than college), and age split by the median (less than or equal to 46/greater than 46).

### Analysis

The goal of the study was to 1) replicate the results of the Ferrer et al. paper (i.e. validate the TRIRISK model for cancer in our sample and compare the TRIRISK model to other single and dual models); 2) explore the TRIRISK model for respiratory illness; and 3) compare the model fit across different groups (demographic factors and tobacco use).

Frequencies, T-tests, and non-parametric χ2 tests in SPSS (IBM, Armonk, NY) were used to compare the demographic and behavioral profile of participants in the current study to that of the participants in the Ferrer et al. sample.

In order to validate the TRIRISK model in our sample, Cronbach’s alpha tests for each putative latent TRIRISK factor in each disease model were performed in SPSS. We also assessed internal consistency, followed by Confirmatory Factor Analyses (CFA) using Mplus version 8.6 (Muthen & Muthen). The first model run directly reproduced the original TRIRISK model (Ferrer et al., 2016), for both respiratory illness and cancer items, respectively. Similar to the Ferrer et al. paper, we used nested χ2 tests to evaluate the *unmodified* TRIRISK model against a single-factor model and two dual-factor models where affective and experiential risk perceptions were consolidated into a single factor, and deliberative and experiential risk perceptions were consolidated. Model fit was evaluated using multiple fit indices. Comparative fit index (CFI) > .95 (or a root mean square error of approximation, RMSEA, < .06) and a standardized root mean square residual (SRMR) < .08 were indicative of good fit to the data (Hu & Bentler, 1999). Modification indices were used iteratively to improve model fit as needed (final models). Finally, in order to compare the TRIRISK model fit across demographic and behavioral groups, multiple group model tests comparing participants who reported being male/female, being White or other, having a college degree or more, or being a current, former, or never smoker were conducted. Nested χ2 tests were used to compare a model with factor loadings constrained to be equal across groups compared to a model without equality constraints.

## Results

### Descriptive statistics

Table 1 shows a breakdown of participants in this study. The mean age of participants was 46.9, the majority reported being female (58.5%), most had a college degree or more (66.9%), and most were White (81.7%). The mean age of our participants was significantly higher compared to the study population in studies 1 and 2 in the Ferrer et al. paper (*p* < 0.0001). The reported percentages of males in our population were significantly lower compared to the study population in studies 1 and 2 in the Ferrer et al. paper (*p* < 0.0001). The percentage of our participants in both education groups were significantly different from the study population in studies 1 and 2 in the Ferrer et al. paper (*p* < 0.0001). Regarding race, the percentage of participants in our sample who were White was significantly higher than the frequencies found in studies 1 and 2 in Ferrer et al. paper (*p* < 0.0001).

**Table 1.** Demographic breakdown of sample used in this study and comparison of this study population to that of the Ferrer et al. paper (Ferrer et al., 2016).

	*N* (%)	Ferrer et al. cancer study 1	*p*-value	Ferrer et al. cancer study 2	*p*-value
Total	704	458		473	
Mean age (SD)	46.93 (16.90)	33.46 (10.07)	<0.0001	32.80 (10.90)	<0.0001
Age range	18–100	18–75		18–67	
Age			–		–
Less than 46	358 (51%)				
46 and Greater	346(49%)				
Sex			<0.0001		<0.0001
Male	278 (40%)	55%		57%	
Female	412 (59%)	45%		43%	
Education			<0.0001		<0.0001
Less than college	233 (33%)	64%		65%	
College or more	471 (67%)	36%		35%	
Race			<0.0001		<0.0001
White	575 (82%)	67%		62%	
Other	129 (18%)	33%		38%	
Smoking status			–		–
Current	65 (9%)				
Former	174 (25%)				
Never	464 (66%)				

### Comparing the TRIRISK model to other model structures

For both cancer and respiratory illness, the single factor models and the two dual factor structures led to a significant decrement in model fit when compared to the respective unmodified TRIRISK models. Table 2 below shows the model fit of each of these models and the results of the nested Chi square tests.

**Table 2.** Model fit indices for final TRIRISK models, original TRIRISK models, one factor models, and two factor models.

	CANCER						RESPIRATORY ILLNESS					
	χ2	df	*p*	CFI	RMSEA (95% CI)	SRMR	χ2	df	*p*	CFI	RMSEA (95% CI)	SRMR
Final model	694.22	124	0.00	0.95	0.08 (0.08, 0.09)	0.05	795.95	127	0.00	0.96	0.09 (0.08, 0.09)	0.04
Original TRIRISK	1857.92	132	0.00	0.85	0.14 (0.13, 0.14)	0.07	1625.99	132	0.00	0.91	0.13 (0.12, 0.13)	0.04
One factor	3128.93	135	0.00	0.74	0.18 (0.17, 0.18)	0.11	2762.86	135	0.00	0.84	0.17 (0.16, 0.17)	0.70
Two factor 1	2280.80	134	0.00	0.82	0.15 (0.15, 0.16)	0.08	1968.80	134	0.00	0.89	0.14 (0.13, 0.15)	0.05
Two factor 2	2141.32	134	0.00	0.83	0.15 (0.14,0.15)	0.09	1942.63	134	0.00	0.89	0.14 (0.13, 0.14)	0.06

### Final models

#### Cancer

The fit indices of the final and original cancer model can be seen in Table 2. The original TRIRISK model did not include any error covariances (just the factor structure with factor covariances). Overall, the original model did not provide a good fit to the data, and accordingly, we modified the model by adding eight error covariances (see Supplemental Table 1 for the specific covariances). Error covariances do not substantially change the factor structure thus the TRIRISK model was retained. A good model fit is indicated based on the 2-index fit criteria and a nested chi square test demonstrated that the final model had significantly better fit in comparison to the original model. The loadings of indicators for each latent factor of the final models are listed in Table 3.

**Table 3.** Standardized factor loadings of final models.

		CANCER		RESPIRATORY ILLNESS	
Item Number		Coefficient	*p*-value	Coefficient	*p*-value
	**Deliberative**	alpha = 0.73		alpha = 0.76	
2	How likely is it that you will get ___ at some point in the future?	0.84	0.00	0.94	0.00
1	On a scale from 0 to 100%, how would you rate the probability that you will develop ___ in the future?	0.75	0.00	0.87	0.00
3	The way I look after my health means that my odds of getting ___ in the future are:	0.83	0.00	0.88	0.00
4	When I think carefully about my lifestyle, it does seem possible that I could get ___.	0.60	0.00	0.65	0.00
5	If I look at myself as if I was a doctor, I realize that my behavior puts me at risk of getting ___.	0.49	0.00	0.66	0.00
10	How do you think your chance of developing __ in the future compares to the average person of your gender and age?	0.62	0.00	0.71	0.00
	**Affective**	alpha = 0.97		alpha = 0.98	
11	How worried are you about developing ___ in the future?	0.90	0.00	0.97	0.00
12	How fearful are you about developing ___ in the future?	0.95	0.00	0.97	0.00
13	How nervous are you about developing ___ in your lifetime?	0.95	0.00	0.97	0.00
14	When you think about ___ for a moment, to what extent do you feel fearful?	0.85	0.00	0.92	0.00
15	When you think about ___ for a moment, to what extent do you feel worried?	0.88	0.00	0.92	0.00
16	When you think about ___ for a moment, to what extent do you feel anxious?	0.83	0.00	0.90	0.00
	**Experiential**	alpha = 0.56		alpha = 0.61	
17	How concerned are you about developing __ in your lifetime?	0.91	0.00	0.93	0.00
18	How easy is it for you to imagine yourself developing __ future?	0.78	0.00	0.90	0.00
6	I feel very vulnerable to ___.	0.62	0.00	0.78	0.00
7	I am confident that I will not get ___. [reverse scored]	0.54	0.00	0.60	0.00
8	I would be lying if I said ‘There is no chance of me getting ___.’	0.34	0.00	0.34	0.00
9	My first reaction when I hear of someone getting ___ is ‘that could be me someday.’	0.53	0.00	0.71	0.00

#### Respiratory illness

The model fit indices of the final and original respiratory illness model can be seen in Table 2. Similar to the cancer model, the original TRIRISK model did not include any error covariances (just the factor structure and factor covariances). Overall, the original model did not provide a good fit to the data, and accordingly, we modified the model by adding five error covariances (see supplemental Table 1 for the specific covariances). Error covariances do not substantially change factor structure thus the TRIRISK model was retained. A good model fit is indicated based on the 2-index fit criteria and a nested chi square test demonstrated that the final model had significantly better fit in comparison to the original model. The loadings for indicators for each latent factor of the final models are listed in Table 3.

Table 3 shows the standardized factor loadings for final cancer and respiratory illness models along with the Cronbach’s alpha for each latent TRIRISK factor. The alpha values for the latent factors if items were deleted can be seen in supplemental Table 2. All loadings were statistically significant, and with the exception of item number 8, they were all substantial (>.50), suggestive of a strong factor model.

### Testing Final TRIRISK models across groups

The modified versions of the TRIRISK model (with established reasonable fit) for both cancer and respiratory illness were used to run multiple group models for sex, race, education, and smoking status.

#### Cancer

Regarding the factor loadings of the final TRIRISK cancer model across the demographic and behavioral subgroups, χ2 nested model tests indicated that constraining the factor loadings for sex, education, and smoking status led to a significant decrement in model fit (Table 4). These results suggest statistically significant differences in the magnitude of the factor loadings across these groups. To consider if these differences in magnitude had practical implications for the interpretation of the factors, we inspected the ranges of the factor loadings in the unconstrained model across subgroups. There were some discrepancies. Specifically, for **sex**, male and female ranges for the deliberative construct were 0.57-0.86 and 0.42-0.83; ranges for the affective construct were 0.80-0.97 and 0.84-0.97; and ranges for the experiential construct were 0.21-0.87 and 0.42-0.93. The standardized factor loading that was not substantial among the male group was for item 8. Examining the unconstrained model, this item was substantial for females but was not substantial for males. These results suggest that the factor loadings for the TRIRISK model across these groups may be different. For **education**, less than college and college plus respective ranges for the deliberative construct were 0.48-0.85 and 0.50-0.82; ranges for the affective construct were 0.76-0.93 and 0.86-0.97; and ranges for the experiential construct were 0.41-0.89 and 0.31-0.92. The standardized factor loading that was not substantial among the college plus group (<0.35) was for item 8. Examining the unconstrained model, this item was substantial for the less than college group but was not substantial for the college plus group. These results suggest that the factor loadings for the TRIRISK model across these groups may be different. Overall, the basic factor structure of the TRIRISK model was similar across sex and education, with item 8 loading a bit differently across the groups. For **smoking status**, respective current, former, and never ranges for the deliberative construct were −0.11-0.91, 0.57-0.89, and 0.51-0.82; ranges for the affective construct were 0.84-0.98, 0.83-0.95, and 0.80-0.96; and ranges for the experiential construct were 0.40-0.88, 0.38-0.94, and 0.33-0.91. For the deliberative construct, the standardized factor loading for item 5 was not substantial (<0.35) for current smokers but was for former and never smokers. For the experiential construct, item 8 was substantial for current and former smokers, but was not for never smokers. Overall, the factor structure for the TRIRISK model was similar, but some items (5 and 8) loaded differently. The nested test for **race** suggested no decrement in model fit, suggesting the magnitude of the factor loadings was similar across racial groups.

**Table 4.** Comparing TRIRISK model fit across demographic and behavioral groups.

	**CANCER**			**RESPIRATORY ILLNESS**		
	Unconstrained Model	Constrained Model	Modified Constrained Model	Unconstrained Model	Constrained Model	Modified Constrained Model
**Sex**						
Chi square	907.10	947.65	796.44	991.64	1013.12	930.28
DF	263	278	268	269	284	281
Male contribution	365.44	389.37	386.04	449.91	463.01	460.94
Female contribution	541.67	558.28	410.40	541.73	550.11	469.34
**Race**						
Chi square	994.13	1018.96	855.81	1017.04	1047.40	793.39
DF	263	278	269	269	284	274
White contribution	589.92	594.80	431.94	654.94	659.07	405.00
Other contribution	404.21	424.16	423.88	362.10	388.33	388.40
**Education**						
Chi square	922.66	953.85	764.78	1089.41	1115.09	791.97
DF	263	278	267	269	284	273
Less than college contribution	346.42	368.19	369.09	375.34	392.17	391.88
College or more contribution	576.23	585.66	395.69	714.07	722.91	400.09
**Smoking status**						
Chi square	1233.86	1314.09	1184.89	1244.03	1321.70	1079.83
DF	402	432	424	411	441	430
Current contribution	288.03	332.26	333.80	300.11	360.51	352.97
Former contribution	362.82	390.51	394.46	347.39	358.83	360.96
Never contribution	583.00	591.32	456.63	596.54	602.36	365.90

Regarding the factor structure of the final TRIRISK cancer model, examination of the constrained **sex** model revealed a total χ2 = 947.65, where males contributed 389.37 to the χ2 while females contributed 558.28 (Table 4). Therefore, the model seemed to be fitting worse for females. After freeing error covariances in the female group, the model fit appeared to fit similarly in each group (total χ2 = 796.44, male χ2 = 386.04, female χ2 = 410.40). Examination of the constrained **race** model revealed a total χ2 = 1018.96, where White individuals contributed 594.80 to the χ2 while other race individuals contributed 424.16 (Table 4). Therefore, the model seemed to be fitting worse for White individuals. After freeing error covariances in this group, the model appeared to be fitting similarly in each group (total χ2 = 855.81, White χ2 = 431.94, other χ2 = 423.88). Examination of the constrained education model revealed a total χ2 = 953.85, where those with less than college contributed 368.19 to the χ2 while those with college or more contributed 585.66 (Table 4). Therefore, the model seemed to be fitting worse for individuals with a college or more education. After freeing error covariances in this group, the model appeared to be fitting similarly in each group (total χ2 = 764.78, less than college χ2 = 369.09, college plus χ2 = 395.69). Examination of the constrained **smoking status** model revealed a total χ2 = 1314.09, where current smokers contributed 332.258 to the χ2, former smokers contributed 390.51, and never smokers contributed 591.32 (Table 4). Therefore, the model seemed to be fitting worse for never smokers. After freeing error covariances in this group, the model appeared to be fitting similarly in each group (total χ2 = 1184.89, current χ2 = 333.80, former χ2 = 394.458, never = 456.63). **Overall**, the factor structure of the final TRIRISK cancer model appears to fit across the subgroups of interest.

#### Respiratory illness

Regarding the factor loadings of the final TRIRISK respiratory illness model across the demographic and behavioral subgroups, χ2 nested model tests indicated that constraining the factor loadings for race, education, and smoking status led to a significant decrement in model fit (Table 4). These results suggest that the magnitude of the factor loadings are statistically significantly different across these groups. To consider the practical implications of these differences between the factor loadings across subgroups, we further inspected the ranges of the factor loadings in the unconstrained model across subgroups. For **race**, respective ranges for White and Other individuals for the deliberative construct were 0.67-0.94 and 0.52-0.95; ranges for the affective construct were 0.91-0.98 and 0.88-0.97; and ranges for the experiential construct were 0.33-0.92 and 0.32-0.94. All of the factor loadings were substantial and large, with the exception of item 8. However, this exception is true for both subgroups, thus suggesting that that there are no practical differences in standardized factor loadings between racial groups. For **education**, less than college and college plus respective ranges for the deliberative construct were 0.65-0.94 and 0.62-0.95; ranges for the affective construct were 0.89-0.98 and 0.91-0.97; and ranges for the experiential construct were 0.51-0.93 and 0.24-0.92. All of the factor loadings were substantial and large, except for one discrepancy. The standardized factor loading for item 8 for the college plus group was not substantial, but it was substantial for the less than college group. Overall, the basic factor structure of the TRIRISK model was similar across race and education, with item 8 loading a bit differently across education groups. For **smoking status**, respective current, former, and never ranges for the deliberative construct were 0.27-0.96, 0.57-0.95, and 0.64-0.93; ranges for the affective construct were 0.90-0.97, 0.91-0.99, and 0.90-0.98; and ranges for the experiential construct were 0.42-0.97, 0.38-0.90, and 0.32-0.92. All of the factor loadings were substantial and large, except for two discrepancies. The standardized factor loading for item 5 was not substantial for current smokers but was for former and never smokers. Also, the standardized factor loading for item 8 was not substantial for never smokers but was for current and former smokers. Overall, the factor structure for the TRIRISK model was similar, but some items (5 and 8) loaded differently. The nested test for **sex** suggested no decrement in model fit, suggesting the magnitude of the factor loadings was similar across racial groups.

Regarding the factor structure of the final TRIRISK respiratory illness model, examination of the constrained **sex** model revealed a total χ2 = 1013.12, where males contributed 463.01 to the χ2 while females contributed 550.11 (Table 4). Therefore, the model seemed to be fitting worse for females. After freeing error covariances in the female group, the model fit appeared to be fitting similarly in each group (total χ2 = 930.28, male χ2 = 460.94, female χ2 = 469.34). Examination of the constrained **race** model revealed a total χ2 = 1047.40, where White individuals contributed 659.07 to the χ2 while other race individuals contributed 388.33 (Table 4). Therefore, the model seemed to be fitting worse for White individuals. After freeing error covariances in this group, the model appeared to be fitting similarly in each group (total χ2 = 793.39, White χ2 = 405.00, other χ2 = 388.40). Examination of the constrained **education** model revealed a total χ2 = 1115.09, where those with less than college contributed 392.17 to the χ2 while those with college or more contributed 722.91 (Table 4). Therefore, the model seemed to be fitting worse for individuals with a college or more education. After freeing error covariances in this group, the model appeared to be fitting similarly in each group (total χ2 = 791.97, less than college χ2 = 391.88, college plus χ2 = 400.09). Examination of the constrained **smoking status** model revealed a total χ2 = 1314.09, where current smokers contributed 360.51 to the χ2, former smokers 358.83 contributed, and never smokers contributed 602.36 (Table 4). Therefore, the model seems to be fitting worse for never smokers. After freeing error covariances in this group, the model appeared to be fitting similarly in each group (total χ2 = 1079.83, current χ2 = 352.97, former χ2 = 360.96, never = 365.90). **Overall**, the factor structure of the final TRIRISK respiratory illness model appears to fit across the subgroups of interest.

## Discussion

The present study utilized a TRIRISK framework from previous literature (Ferrer et al., 2016) for two different diseases. Broadly, the TRIRISK structure was replicated for cancer and extended adequately to respiratory illness, thereby providing evidence for future use of this model, particularly in tobacco control research The original model did not provide a good fit to the data, and accordingly, we used post hoc modifications by freeing error covariances to improve model fit. Specifically, these modifications did not substantially change the factor structure. Thus, within our population, the TRIRISK model was retained and the 3 distinct risk perception constructs are supported. For cancer and respiratory illness, items loaded on the respective constructs as expected and the internal reliability among deliberative, affective, and experiential components suggested good convergent validity. We also extended this work to examine factorial invariance. In this sample, the TRIRISK model fit for cancer and respiratory illness risk perceptions and was invariant for sex, race, education, and smoking status. The fact that the TRIRISK model framework fits well across differing subgroups suggests that this is a valid model of risk perception to use in a general population sample. Of particular interest is the fact that the TRIRISK model fits well across smoking status groups. Smoking is a known high-risk factor for both cancer and respiratory illnesses (Centers for Disease Control and Prevention, 2020). These results provide further evidence that the TRIRISK model is a useful model for risk perceptions in tobacco control research.

Regarding the standardized factor loadings across different subgroups of interest, there were few discrepancies in magnitude. The few discrepancies could be explained by interindividual differences in item response. For example, people who currently smoke and formerly smoked may perceive their risk differently compared to people who have never smoked. Ferrer et al. found that firsthand experience with a disease leads to both more differentiated and higher perceptions of deliberative, affective, and experiential risk (Ferrer et al., 2016).

Compared to the Ferrer and colleagues’ paper, there was some indication that internal reliability in this sample was lower (Cronbach’s alpha values ranged from 0.56–0.97). The latent factor correlations found here (deliberative-affective 0.70; deliberative-experiential 0.81; affective-experiential 0.95) were higher than those reported by Ferrer and colleagues (Ferrer et al., 2016), which may indicate lower discriminant validity. However, model fit worsened significantly when the constructs were collapsed (single and dual models led to a significant decrement in model fit compared to the TRIRISK model), suggesting they are distinct. The only model modifications made were freeing some error covariances (a somewhat different approach than the Ferrer et al. studies, but theoretically justifiable (McDonald & Ho, 2002)). Overall, the TRIRISK pattern seems to hold in both of these diseases, and our analysis supports risk perception as a multidimensional construct.

There are many strengths to our study, including a relatively large sample size drawn from the general population. In addition to replicating the TRIRISK model in cancer, it was extended to another disease, respiratory illness, providing additional conceptual validation of the framework. Lastly, our data collection methods included random address sampling and mail-based invitations, which helps in widening the population sampling frame. We also addressed a key limitation mentioned in the Ferrer et al. paper – their relatively low indices of goodness-of-fit, which limited their interpretation of the findings. In our sample, we obtained good model fit based on a 2-index criteria (Hu & Bentler, 1999) by freeing error covariances.

The limitations of our study include that the data collection occurred during the COVID-19 pandemic. This may have impacted how individuals responded to risk perception questions around respiratory illness. Furthermore, our questions did not include ‘don’t know’ response options, as in the Ferrer et al. study, which may have been meaningful and could have affected our results. Although the data were correlational, this approach is appropriate because we were attempting to validate and explore the model in specific diseases. Furthermore, changing risk perceptions has been causally linked to behavior change in previous research (Ferrer et al., 2016). Another weakness of this study, as in the Ferrer et al. study, was that the test-retest reliability was not examined and would be beneficial with regards to the accuracy of the TRIRISK scales. Finally, although we found that fit for the tripartite model was significantly better than the fit for the single or dual-factor models, we used unmodified versions of each, and therefore no error covariances were allowed, regardless of their modification index. However, since the unmodified models demonstrated worse fit then the modified models, the results suggest that the TRIRISK model would be better even after modifying the models based on a certain criterion.

Overall, our sample somewhat provides additional empirical evidence in support of the TRIRISK model, replicating the factor structure for cancer risk perceptions. Our sample also extends the TRIRISK model to respiratory illness risk perceptions. Future research could look in further detail at the differences between the three factors in validated diseases and can examine individual characteristics (e.g. disease knowledge, numeracy, health motivation, socioeconomic status (Ferrer et al., 2016)) to understand these differences. Because some of the standardized factor loadings were close to or over 0.90, some items may be redundant, suggesting that the measure could be streamlined. Future studies guided by item response theory could assess whether items could be consolidated to construct a briefer measure. Although our intention was to replicate the TRIRISK model as specified by Ferrer et al, alternative structures are possible. For example, a bifactor model could be used to specify an overall risk perception factor, and then three orthogonal domain specific risk perception factors. Extending the TRIRISK model to other diseases that may not share specific domains of risk perception may require an EFA approach to determine the appropriate factor structure. We did not test the predictive validity of the TRIRISK model for respiratory illness. Future studies should adopt a prospective design to elucidate the appropriate pathway direction to examine whether or not the TRIRISK model predicts engagement in health behaviors, such as screening and smoking cessation.

Identifying and validating risk perception models are pivotal for informing policies and educational campaigns to demonstrate how health promotion practitioners and medical professionals should design risk communications and decision aids (Ferrer et al., 2016). The appropriate combination of deliberative, affective, and experiential risk perceptions for a specific population could be found for a particular disease and subsequently targeted using tailored messaging. Elaborating the TRIRISK model is an important step to understanding and improving risk communication, as well as encouraging positive health behaviors in populations.

## Supplementary Material

Supplemental Material

## Data Availability

The data that support the findings of this study are available on request from the corresponding author. The data are not publicly available due to privacy or ethical restrictions.
