# Supplementary material for: Exploring the psychometric properties of a tripartite model of risk perception (TRIRISK) in a general U.S. population sample
Source: Health Psychol Behav Med. 2022 Nov 10;10(1):1110–23. doi: 10.1080/21642850.2022.2143363 (PMC9662002; doi:10.1080/21642850.2022.2143363)
Supplement: Supplemental Material [file RHPB_A_2143363_SM4529.docx]

**SUPPLEMENTAL**

**Table 1.** Standardized covariance estimates for the final cancer and respiratory illness models.

| **CANCER** | | | | | | | |
| --- | --- | --- | --- | --- | --- | --- | --- |
| Item Numbers | | Coefficient | p-value | Latent Factor | | Coefficient | p-value |
| 15 | 14 | 0.76 | 0.00 | AFFECTIVE | DELIBERATIVE | 0.70 | 0.00 |
| 15 | 16 | 0.70 | 0.00 | EXPERIENTIAL | DELIBERATIVE | 0.81 | 0.00 |
| 2 | 1 | 0.59 | 0.00 | EXPERIENTIAL | AFFECTIVE | 0.95 | 0.00 |
| 16 | 14 | 0.60 | 0.00 |  |  |  |  |
| 5 | 4 | 0.33 | 0.00 |  |  |  |  |
| 5 | 6 | 0.27 | 0.00 |  |  |  |  |
| 8 | 7 | 0.23 | 0.00 |  |  |  |  |
| 7 | 6 | 0.20 |  |  |  |  |  |
| **RESPIRATORY ILLNESS** | | | | | | | |
| Item Numbers | | Coefficient | p-value | Latent Factor | | Coefficient | p-value |
| 16 | 15 | 0.70 | 0.00 | AFFECTIVE | DELIBERATIVE | 0.85 | 0.00 |
| 16 | 14 | 0.59 | 0.00 | EXPERIENTIAL | DELIBERATIVE | 0.93 | 0.00 |
| 5 | 4 | 0.41 | 0.00 | EXPERIENTIAL | AFFECTIVE | 0.96 | 0.00 |
| 14 | 11 | -0.13 | 0.00 |  |  |  |  |
| 14 | 15 | 0.61 | 0.00 |  |  |  |  |

**Table 2.** Alpha values for latent factors if items are deleted.

|  | **Cancer** | **Respiratory Illness** |
| --- | --- | --- |
| item | **Deliberative** | |
|  | alpha=0.73 | alpha=0.756 |
| 2 | 0.525 | 0.567 |
| 1 | 0.568 | 0.607 |
| 3 | 0.61 | 0.609 |
| 4 | 0.751 | 0.779 |
| 5 | 0.752 | 0.781 |
| 10 | 0.75 | 0.782 |
|  | **Affective** | |
|  | alpha=.97 | alpha=0.983 |
| 11 | 0.967 | 0.981 |
| 12 | 0.96 | 0.98 |
| 13 | 0.96 | 0.979 |
| 14 | 0.96 | 0.98 |
| 15 | 0.957 | 0.979 |
| 16 | 0.963 | 0.981 |
|  | **Experiential** | |
|  | alpha=0.555 | alpha=0.61 |
| 17 | 0.165 | 0.2 |
| 18 | 0.161 | 0.189 |
| 6 | 0.562 | 0.615 |
| 7 | 0.564 | 0.62 |
| 8 | 0.568 | 0.626 |
| 9 | 0.563 | 0.617 |
